# Comprehensive characterization of T-DNA integration induced chromosomal rearrangement in a birch T-DNA mutant

**DOI:** 10.1186/s12864-019-5636-y

**Published:** 2019-04-23

**Authors:** Huixin Gang, Guifeng Liu, Manman Zhang, Yuming Zhao, Jing Jiang, Su Chen

**Affiliations:** State Key Laboratory of Tree Genetics and Breeding, Northeast Forestry University, 26 Hexing Road, Harbin, 150040 China

**Keywords:** T-DNA integration, Multiple T-DNA, Chromosomal rearrangement, T-DNA mutant, Birch

## Abstract

**Background:**

Integration of T-DNA into plant genomes via *Agrobacterium* may interrupt gene structure and generate numerous mutants. The T-DNA caused mutants are valuable materials for understanding T-DNA integration model in plant research. T-DNA integration in plants is complex and still largely unknown. In this work, we reported that multiple T-DNA fragments caused chromosomal translocation and deletion in a birch (*Betula platyphylla × B. pendula*) T-DNA mutant *yl*.

**Results:**

We performed PacBio genome resequencing for *yl* and the result revealed that two ends of a T-DNA can be integrated into plant genome independently because the two ends can be linked to different chromosomes and cause chromosomal translocation. We also found that these T-DNA were connected into tandem fragment regardless of direction before integrating into plant genome. In addition, the integration of T-DNA in *yl* genome also caused several chromosomal fragments deletion. We then summarized three cases for T-DNA integration model in the *yl* genome. (1) A T-DNA fragment is linked to the two ends of a double-stranded break (DSB); (2) Only one end of a T-DNA fragment is linked to a DSB; (3) A T-DNA fragment is linked to the ends of different DSBs. All the observations in the *yl* genome supported the DSB repair model.

**Conclusions:**

In this study, we showed a comprehensive genome analysis of a T-DNA mutant and provide a new insight into T-DNA integration in plants. These findings would be helpful for the analysis of T-DNA mutants with special phenotypes.

**Electronic supplementary material:**

The online version of this article (10.1186/s12864-019-5636-y) contains supplementary material, which is available to authorized users.

## Background

*Agrobacterium tumefaciens* can integrate a transfer DNA (T-DNA) into plant genomes. T-DNA is considered as a part of tumor-inducing plasmid from right border (RB) to left border (LB) [1]. Up to now, *Agrobacterium-*mediated T-DNA transformation has been widely used as a powerful tool to generate transgenic materials in plant research [2–4]. The integration of T-DNA into plant genomes has also been used to generate mutant libraries in rice [5], *Arabidopsis* [6] and tobacco [7]. Two models have been suggested for T-DNA integration, including single-stranded gap repair and double-stranded break (DSB) repair [8]. However, the single-stranded gap repair model cannot account for the formation of several complex T-DNA integration patterns. Recently, more and more researches provide evidences to support the DSB model. Tzfira et al. proved that T-DNA could be converted to double-stranded intermediates before being inserted into the DSB sites of the plant genome [9]. Kleinboelting et al. analyzed thousands of T-DNA insertion sites (ISs) in *Arabidopsis* and found that the majority of the ISs are consistent with the DSB repair model [10].

In recent years, chromosomal translocations induced by T-DNA integration has been observed in many T-DNA transformants [11–14]. While the integrations of T-DNA also result in other large chromosomal rearrangements including deletion, duplication and inversion. Tax et al. described that duplication of genomic DNA induced by T-DNA were inserted to another chromosomal locus in two T-DNA lines [15]. Zhu et al. reported two tandem copies of T-DNA insertion caused an inversion of a 4.9-Mb chromosomal segment in a transgenic rice line [16]. In addition, an *Arabidopsis* mutant, *seb19* was even observed to produce translocation, inversion, deletion and duplication on chromosomes 4 and 5 evoked by T-DNA integration [17].

It was known that the RB and LB of a T-DNA were integrated into the plant genome with filler DNA or deletions at the junctions between T-DNA and plant genomic DNA [18]. However, insertions of truncated or rearranged T-DNA fragments into the plant genome occurred frequently. Truncated T-DNA with part of T-DNA fragment missing was observed in the analysis of tobacco transgenic plants [19]. Lechtenberg et al. found two RB fragments directly linked to each other in *Arabidopsis* T-DNA transformants [20]. In a rice T-DNA insertion population, 12,948 flanking sequence tags (FSTs) were isolated from 63,000 transgenic lines by thermal asymmetric interlaced PCR (TAIL)-PCR and 8840 (68.3%) FSTs were mapped to the rice genome [21]. The other FSTs were T-DNA repeat, vector sequences or non-specific amplification. Hence, T-DNA integration is a complex process and largely remain unknow. Previous researches of chromosomal translocations or other chromosomal variations induced by T-DNA in *Arabidopsis* or rice were incomplete in above one or more respects. However, multiple T-DNA fragments caused chromosomal translocation and deletion have not reported.

Most likely, an effective way for clarifying T-DNA integrations into plant genomes is the large-scale analysis of ISs in T-DNA lines. Currently, with the development of sequencing technology, new sequencing technologies such as Next generation sequencing (NGS) and Pacific Biosciences RS (PacBio) sequencing are widely used for genomic biology analysis [22]. NGS provides high-throughput data with high accuracy and has been widely applied in whole genome sequencing [23, 24]. However, the NGS reads are too short to resolve abundant repeats or other complex genomic regions. The recent arrival of PacBio contributes in producing long reads that exceed 60 kb, which is complement to the NGS technology. The PacBio sequencing would be helpful for genome research of chromosomal rearrangement induced by T-DNA integration in mutants.

Here, we found a T-DNA mutant *yl* had less viable pollens and seeds. The *yl* mutant was derived from the transgenic lines overexpressing *BpCCR1* gene. However, the pollens and seeds of all the transgenic birch plants expect *yl* displayed normal phenotype like the wild type birch (WT). The overexpression plasmid vector pGWB2-*BpCCR1* was constructed using the full-length cDNA sequence of *BpCCR1* gene with the cauliflower mosaic virus promoter. These transgenic lines were generated using *Agrobacterium*-mediated transformation and they were heterozygous for T-DNA integration. We performed PacBio sequencing for *yl* to dissect the possible reason of the phenotype. We found that complex chromosomal rearrangements, including translocation and deletion, induced by T-DNA occurred in the *yl* genome. These chromosomal structural variations may be responsible for the unique phenotype. We then discuss the model for T-DNA integration based on the model previous reported. Our results provide a better understanding of the integration of T-DNA into plant genome by DSB repair model.

## Results

### Phenotypes of the gametes and seeds in a T-DNA insertion mutant

**Fig. 1:**
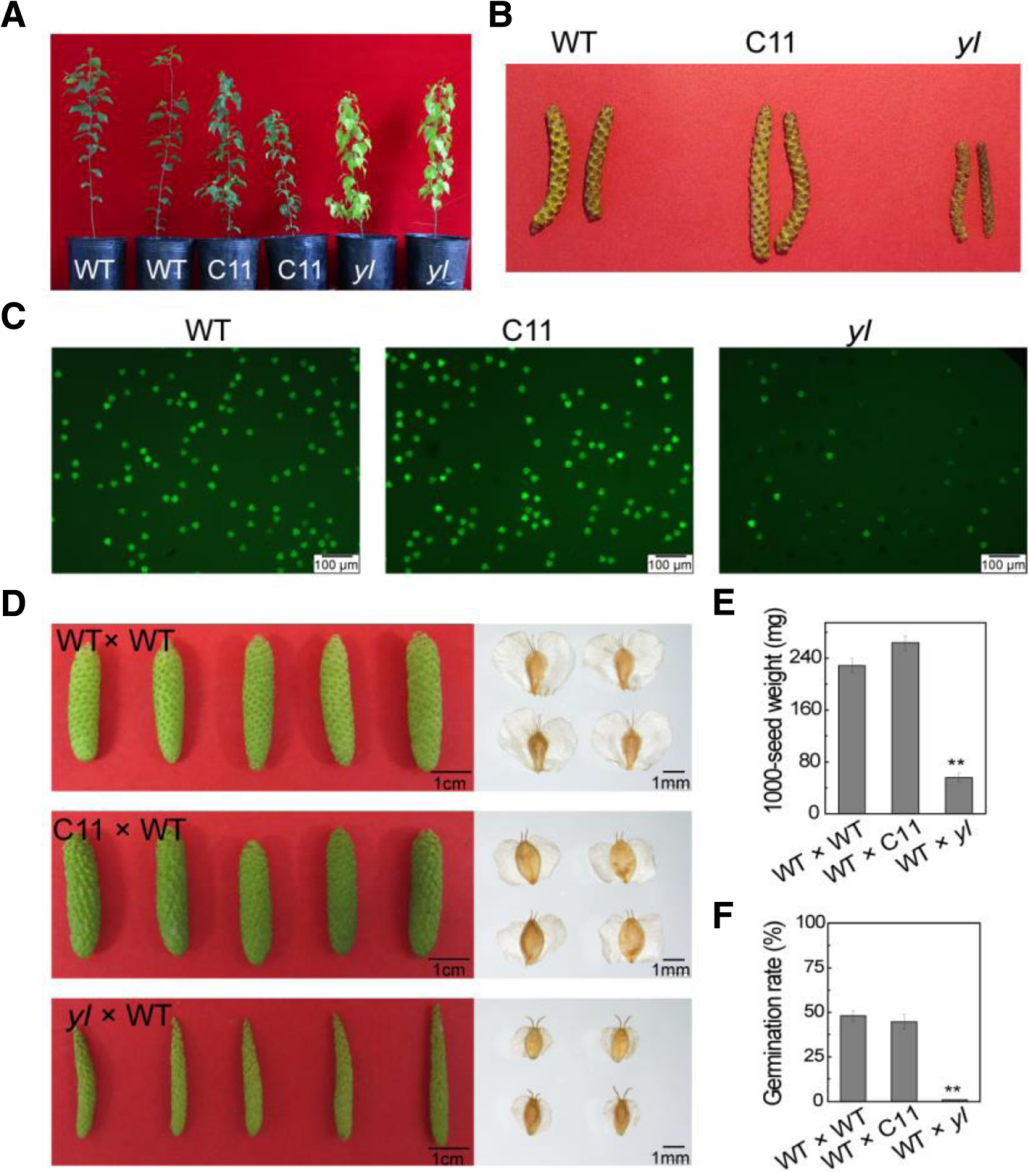
Phenotypes of the gametes and seeds in *yl*. **a** Isolation of a *yl* mutant from *BpCCR1* transgenic lines. WT, wild type birch. C11 and *yl* were the *BpCCR1* overexpression lines. **b** Phenotypes of male inflorescence in WT, C11 and *yl*. c Fluorescein diacetate staining for pollen viability in WT, C11 and *yl*. Scale bars = 100 μm. **d** Phenotypes of infructescences and seeds in cross-combinations of WT × WT, C11 × WT and *yl* × WT. **e** 1000-seed weight of WT × WT, C11 × WT and *yl* × WT seeds. Scale bars = 1 cm (left) and 1 mm (right). **f** Germination rate of WT × WT, C11 × WT and *yl* × WT seeds. ** indicated *P* value < 0.01 (t test). Error bars represent the SD of three independent experiments

In our previous study, we isolated a T-DNA yellow-green mutant *yl* form 19 *BpCCR1* (cinnamoyl-CoA reductase) overexpression transgenic lines [25]. We used the wild type birch and another *BpCCR1* transgenic line C11 as the control plants (Fig. 1a). Besides leaf-color, we found that the male inflorescences of *yl* were stunted and smaller compared to WT and C11 (Fig. 1b). We then examined the viability of pollens collected from WT, C11 and *yl* plants, and found that most of the pollens from *yl* were non-viable compared to control lines (Fig. 1c). To examine the viability of seeds in *yl*, we performed hybridization experiment using *yl* as female parent and WT as the male parent (*yl* × WT). The infructescences and seeds from cross-combination of *yl* × WT were smaller than cross-combinations of WT × WT and C11 × WT (Fig. 1d). The 1000-seed weight of *yl* × WT were lighter compared to WT × WT and C11 × WT (Fig. 1e). The germinating rate of the seeds was quite low, only about 1%. However, the germinating rate of seeds collected form WT and C11 were about 48 and 45%, respectively (Fig. 1f). All these observations revealed that *yl* produced very little viable gametes.

### Chromosomal translocations induced by T-DNA integrations in *yl* genome

**Fig. 2:**
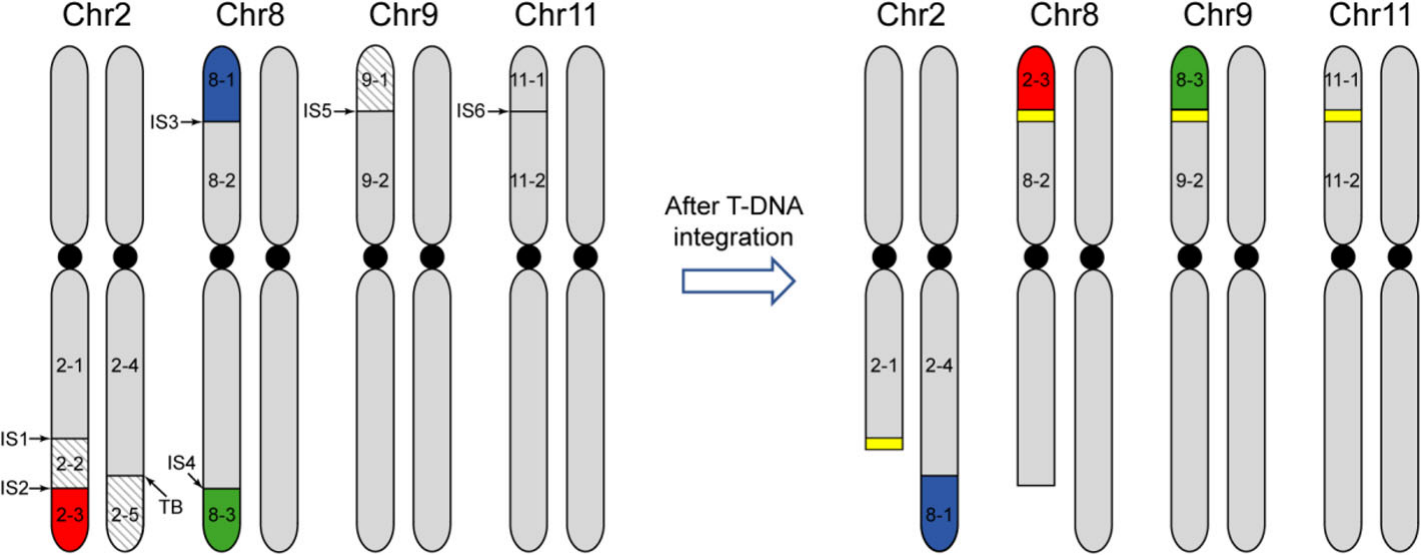
Schematic diagram of chromosomal rearrangement induced by T-DNA in *yl*. Chr2–3 (red), Chr8–1 (blue) and Chr8–3 (green) were translocated fragments. Chr2–2, Chr2–5 and Chr9–1 in diagonal stripe pattern were the deletion regions. T-DNA was marked in yellow

We identified six T-DNA insertion sites including Chr2 23,466,399 (IS1), Chr2 26,269,259 (IS2), Chr8 5168,622 (IS3), Chr8 17,725,909 (IS4), Chr9 1,671,992 (IS5) and Chr11 9,184,715 (IS6) in the *yl* genome by NGS sequencing. However, the NGS reads are too short to display the potential chromosomal variations caused by T-DNA integrations. PacBio reads are powerful to reveal the complete scenario of T-DNA integration in the *yl* genome. We further performed PacBio sequencing for *yl* to explore the T-DNA integrations in more detail. We extracted all the reads around the ISs, and found that the T-DNA integrations caused a serious chromosomal chaos of *yl* genome referring to three chromosomes. *yl* chromosomes were split into several fragments during the T-DNA integration (Fig. 2). Chr2 was split into Chr2–1, Chr2–2, Chr2–3, Chr2–4 and Chr2–5. Chr8 was split into Chr8–1, Chr8–2 and Chr8–3. Chr9 was split into Chr9–1 and Chr9–2. Chr11 was split into Chr11–1 and Chr11–2. Only Chr11–1 and Chr11–2 were properly reconnected after T-DNA integration, while all the other chromosomal fragments were chaotically connected. Chr2–3 was connected to Chr8–2, and Chr8–3 was connected to Chr9–2, respectively. Moreover, we found that Chr8–1 was translocated to Chr2 26,227,384 (translocation breakpoint, TB) without any T-DNA sequences (Fig. 2). Pollen viability was considered to be an estimator of chromosomal translocations occurred in T-DNA lines [26]. Thus, these chromosomal translocations in *yl* might lead to the low gametes viability.

### Multiple T-DNA fragments linked to different chromosomal fragments

**Fig. 3:**
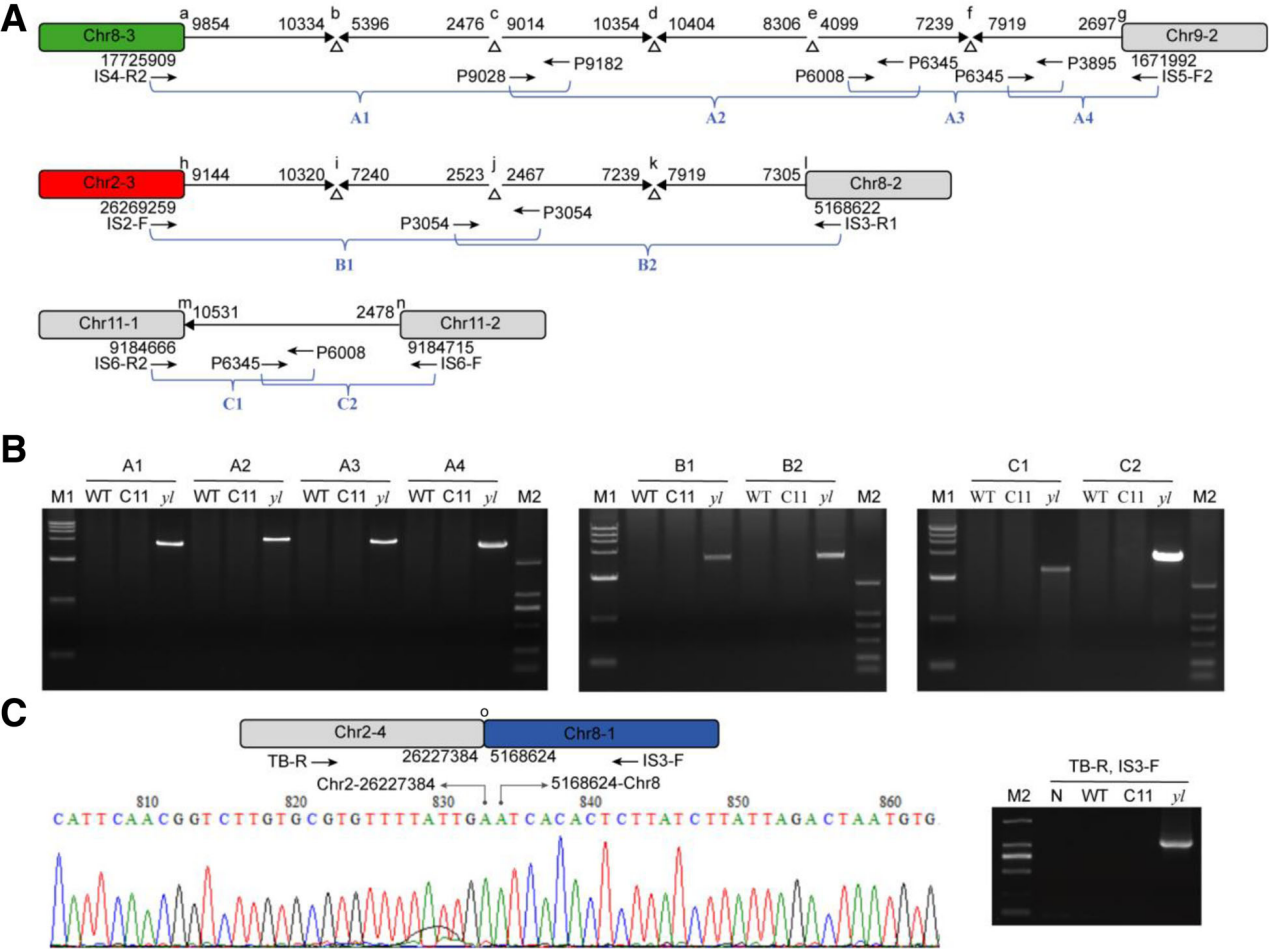
Schematic diagram and verification of the new junctions induced by T-DNA integration in the *yl* genome. **a** Schematic diagram of the primers’ location around the new junctions. Letters a to o represent the new junctions in the *yl* genome. **b** Gel electrophoresis of PCR amplification products. M1, DNA Maker DL15000. M2, DNA Maker DL2000. A1, PCR with primer IS4-R2 and P9182. A2, PCR with primer P9028 and P6345. A3, PCR with primer P6008 and P3895. A4, PCR with primer P6345 and IS5-F2. B1, PCR with primer IS2-F and P3054. B2, PCR with primer P3054 and IS3-R1. C1, PCR with primer IS6-R2 and P6008. C2, PCR with P6345 and IS6-F. **c** Verification of the junction between Chr2–4 and Chr8–1. Gel electrophoresis showed PCR amplification products with primer TB-R and IS3-F. N. Negative control (water)

We found that only IS6 in Chr11 was inserted with an intact T-DNA fragment (from RB to LB) and lead to a short deletion around IS6. This intact T-DNA integration into *yl* genome did not cause chromosomal translocation. However, T-DNA fragments linked to each other were detected to be caused chromosomal translocations (Fig. 3a). T-DNA left border of pGWB2 was considered to be the sequences from 10,509 to 10,524 and the right border was from 2454 to 2478. However, most breakpoints of these T-DNA fragments were not the left or right border regions. The junction of Chr2–3 and Chr8–2 consisted of four T-DNA fragments, from 9144 to 10,320, 7240 to 2523, 2467 to 7239 and 7919 to 7305, respectively. The junction of Chr8–3 and Chr9–2 was comprised of six T-DNA fragments, from 9854 to 10,334, 5396 to 2476, 9014 to 10,354, 10,404 to 8306, 4099 to 7239 and 7919 to 2697, respectively (Fig. 3a). Normally, the T-DNA of pGWB2 was a fragment of 8031–8080 bp in length. However, the two tandem T-DNA in *yl* were fragments of 15,200 bp and 11,279 bp in length, respectively. The longer T-DNA fragment may be helpful for the generation of chromosomal translocation in *yl*. The results indicated that *Agrobacterium*-mediated T-DNA integration is a complex process. The formation of tandem T-DNA may be the source of chromosomal translocation.

### PCR verification of PicBio sequencing

In order to verify the chromosomal translocation and multiple tandem T-DNA fragments in the *yl* genome, we designed a series of primers to amplify the connections of Chr8–3 to Chr9–2, Chr2–3 to Chr8–2, Chr11–1 to Chr11–2 and Chr2–4 to Chr8–1 (Fig. 3a and c, Additional file 1: Table S3). PCR for large fragment is a challenge. We tried to amplify the connections of Chr8–3 to Chr9–2, Chr2–3 to Chr8–2 and Chr11–1 to Chr11–2 directly, but failed. Therefore, the connection between Chr8–3 and Chr9–2 was divided into four fragments, including A1 to A4. The connection between Chr2–3 and Chr8–2 was divided into two fragments, B1 and B2. The connection between Chr11–1 and Chr11–2 was divided into two fragments, C1 and C2. As shown in Fig. 3b and c, all these fragments could be successfully amplified in the *yl* genome, while not in the WT and C11 genome.

**Fig. 4:**
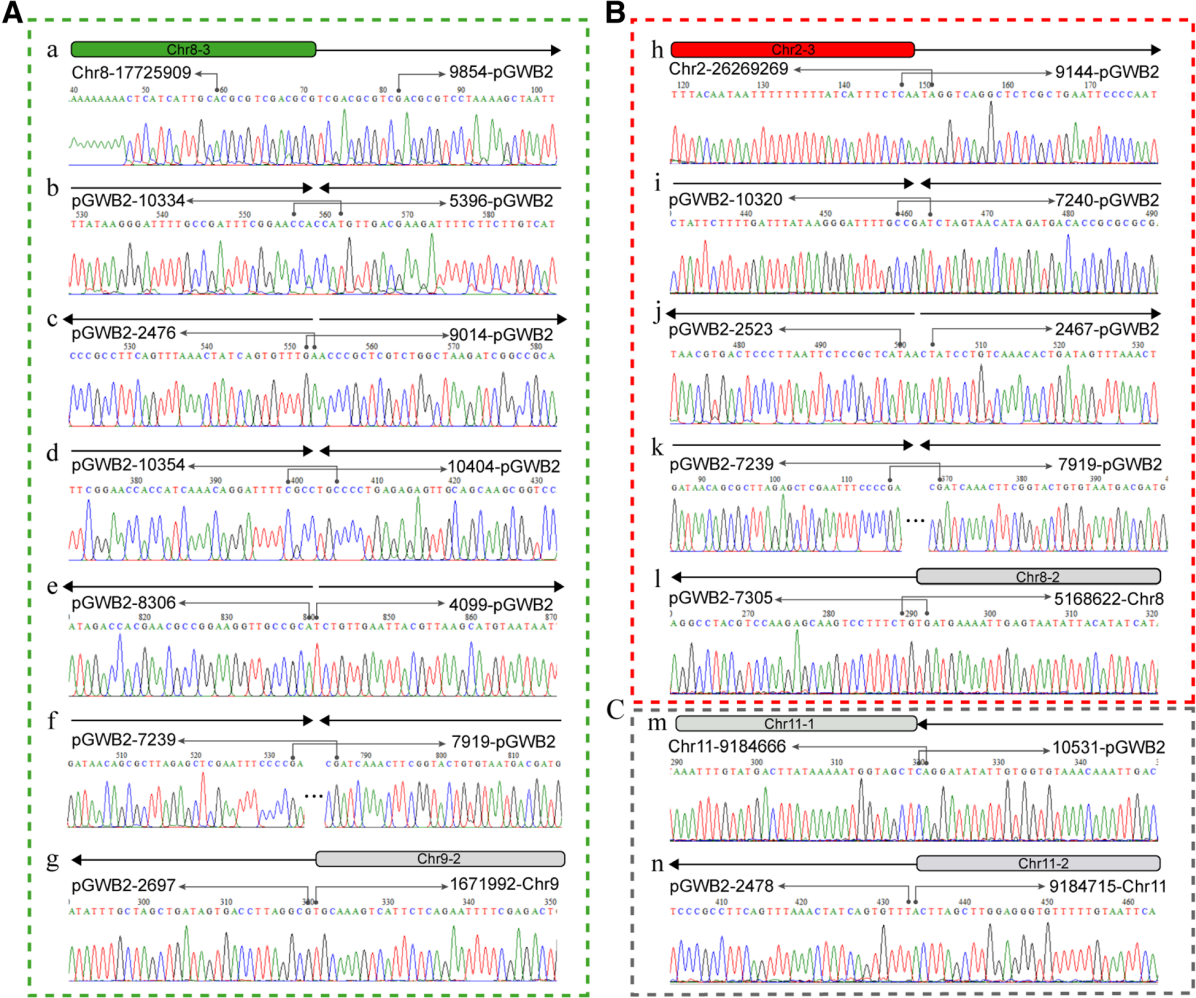
Sanger sequencing of the new junctions a to n in the *yl* genome. **a** The connection between Chr8–3 and Chr9–2. **b** The connection between Chr2–3 and Chr8–2. **c** The connection between Chr11–1 and Chr11–2

We then designed primers to amplify all the junctions (a to o) caused by T-DNA integration. The results revealed that all the junctions have been successfully amplified in *yl* and sequenced by sanger sequencer (Additional file 1: Figure S2). The sequencing results confirmed that all the new junctions (a to o) determined by PacBio sequencing were existed in the *yl* genome (Figs. 3c and 4, Additional file 1: Table S4). We further analyzed all these new junctions and found that the repair model of T-DNA integration in the *yl* genome was basically consistent with non-homologous end-joining (NHEJ) model. All the junctions except k and f showed little (4–7 bp) or no micro-homology sequence at the two ends. The junction k and f showed 254 bp homologous sequences at the two ends. Moreover, a stretch of 22-bp and 3-bp filler DNA were observed to be inserted at the junction a and j, respectively.

### Several chromosomal fragments were lost in the *yl* genome after T-DNA integration

**Fig. 5:**
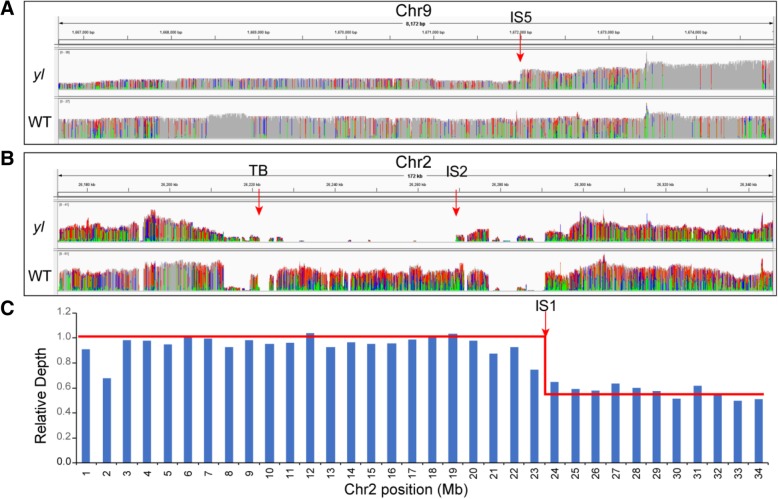
Deletion of chromosomal fragments during chromosomal translocations. **a** Visualized reads coverage of *yl* and WT around IS5 using Integrative Genome Viewer (IGV). **b** Visualized reads coverage of *yl* and WT around IS2 and TB using IGV. **c** Relative Depth of Chr2 with 1-Mb resolution in *yl* compared to WT

Although we identified chromosomal translocations including 3 chromosomes occurred in *yl* according to the resequencing results, we could not trace the new locations of Chr9–1, Chr2–2 and Chr2–5 (Fig. 2). Thus, we speculated that these fragments may be lost during the chromosomal translocation process. In order to confirm our assumption, we mapped the PacBio reads from *yl* and WT to the birch genome and examined the sequencing coverage for each chromosome of *yl* and WT. The sequencing depths for Chr9–1 and Chr9–2 were extracted using “SAMtools depth” function, respectively. We found that the average sequencing depths of Chr9–1 and Chr9–2 in *yl* were 18.6 and 28.4, respectively. However, the average sequencing depths of Chr9–1 and Chr9–2 in WT were 35.6 and 28.5, respectively. Since birch is a diploid species, we speculated that one homologous of Chr9–1 in *yl* may be lost during the T-DNA integration process (Fig. 2). Then we used IGV visualization software to display the reads coverage around IS5. As shown in Fig. 5a, the sequencing coverage of Chr9–1 was halved compared with Chr9–2, which indicated one homologous of Chr9–1was lost from IS5. To our surprise, we found that the regions from IS1 to the end of Chr2 became haploid in *yl* compared to WT (Fig. 5c). We then extracted the reads around TB and found that all the reads were mapped to junction of translocation, but no reads were covered across the region around TB. And the IGV result of Chr2 revealed that the region from breakpoint TB to IS2 (40 kb) was a homozygous deletion (Fig. 5b). These results revealed that the chromosomal fragments Chr9–1, Chr2–2 and Chr2–5 were lost during T-DNA integration and chromosomal translocation.

## Discussion

### T-DNA integration can cause large scale chromosomal structure variations

**Fig. 6:**
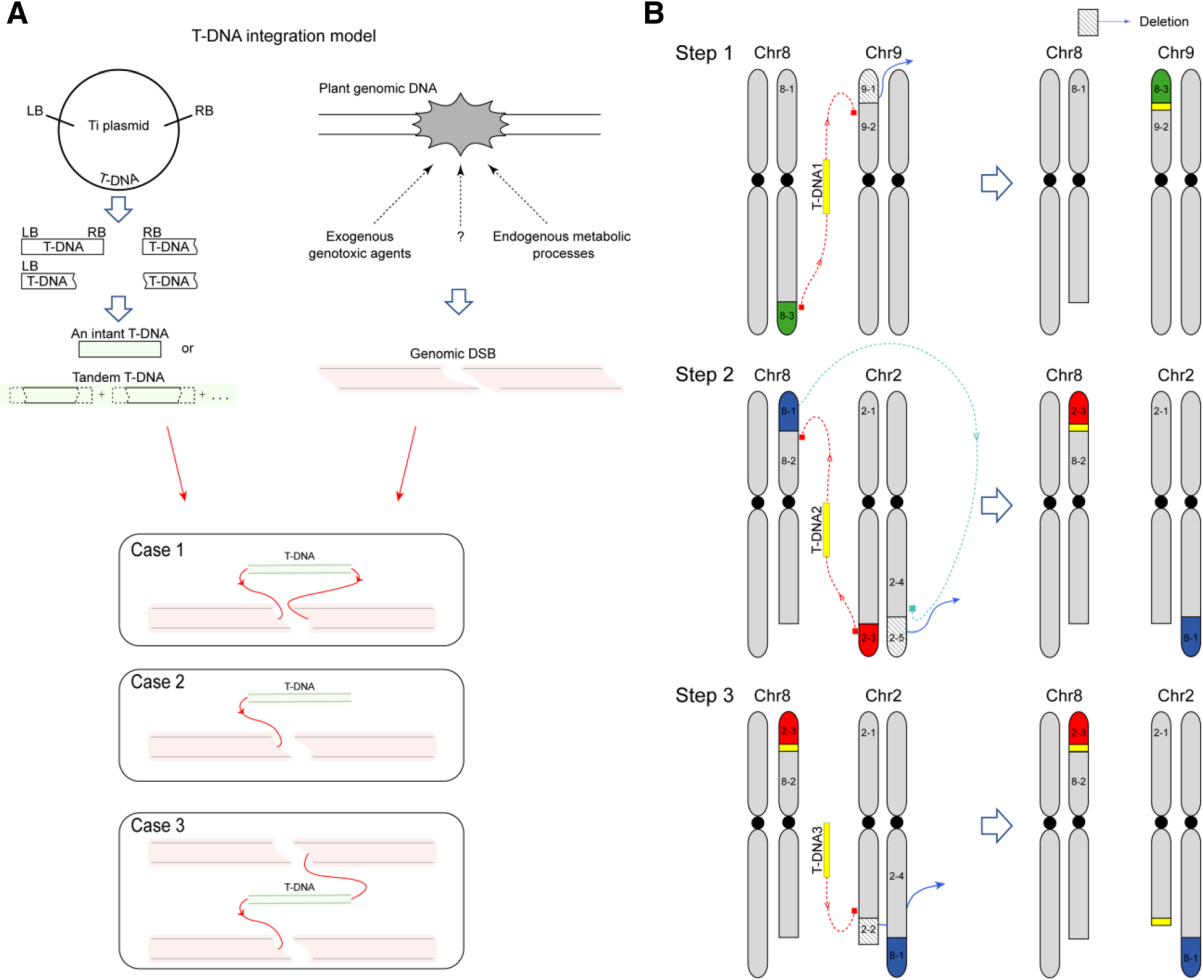
Diagram illustration of T-DNA integration model in the *yl* genome. **a** Overview of the three T-DNA integration models in *yl* genome. **b** Schematic diagram of the three T-DNA integrations which caused chromosomal translocation and/or deletion in the *yl* genome. Step 1 to step 3 indicated three T-DNA integrated into the *yl* genome, independently. T-DNA were marked in yellow. Dashed lines indicate rearrangements and arrows represent the direction of chromosomal rearrangement fragments. Chr8–3 (green), Chr8–1(blue), Chr2–3 (red) were translocated fragments. Chr2–2, Chr2–5 and Chr9–1 in diagonal stripe pattern were the deletion fragments

In eukaryotic cells, the endogenous free radicals from metabolic processes and exogenous genotoxic agents such as ionizing radiation can induce DSBs in genome [27]. Recently, DSB model has been considered for T-DNA integration [10], and the integration in plants is mainly repaired via NHEJ pathway [28]. In this work, we analyzed the genome variations of a birch T-DNA mutant *yl* caused by T-DNA integration. Complex chromosomal translocations and deletions were found in the *yl* genome, when we analyzed the T-DNA ISs of *yl* (Fig. 2). The T-DNA integration in the *yl* genome is consistent with the DSB and NHEJ repair model (Fig. 6). NHEJ rejoins DNA ends with little or no sequence homology [29]. We found that most junctions of T-DNA and plant genome in *yl* also showed little or no micro-homology sequence at the two ends.

T-DNA integration model in the *yl* genome could be summarized into three cases (Fig. 6a). (1) A T-DNA fragment is linked to the two ends of the a DSB; (2) Only one end of a T-DNA fragment is linked to a DSB; (3) A T-DNA fragment is linked to the ends of different DSBs. Only in the first case, a T-DNA fragment is properly inserted into a plant genome, such as IS6 in Chr11 (Figs. 2 and 3). In the second case, T-DNA integration may generate truncated chromosomes. In the *yl* mutant, only one end of T-DNA3 was linked with Chr2–1, and a truncated chromosomal fragment, Chr2–1, was generated (Figs. 2 and 6b). In the third case, the two ends of a T-DNA fragment were linked to different chromosomes, indicating that the integration of the two ends of a T-DNA may be independent. This may cause chromosomal translocations and/or deletion of chromosomal fragments. Chromosomal translocation caused by T-DNA heterogeneous integration like this had also been reported in *Arabidopsis seb*19 mutant [17]. In *yl,* T-DNA1 was linked to Chr9–2 and Chr8–3 independently, and as a consequence, Chr9–1 was lost (Fig. 6b). Similarly, the two ends of T-DNA2 were connected with Chr2–5 and Chr8–2, respectively, and the dissociated Chr8–1 linked to Chr2–1, which lead to the deletion of Chr2–2 (Fig. 6b). Moreover, we found that the T-DNA whose two ends linked to different chromosomes were comprised of multiple T-DNA fragments (Fig. 3). Chr8–3 and Chr9–2 were connected through six parts of T-DNA fragments. Chr2–5 and Chr8–2 were connected through four T-DNA fragments. The tandem T-DNA linked to each other regardless of their respective directions or length. The insertions of more T-DNA fragments may provide extended regions for heterogeneous integration in plant genome, and these are consistent with the DSB repair model.

### Phenotype of *yl* can be explained by complex chromosomal translocation

Clark et al. found chromosomal translocation is a common phenomenon in *Arabidopsis* T-DNA transformants. In our research, we only detected one line *yl* among the 19 *BpCCR1* overexpression lines contained chromosomal translocation. In *Arabidopsis*, the frequency of chromosomal translocation in the transgenic lines was estimated to be about 19%. And many abnormal pollens were observed in most of these lines with chromosomal translocation [26]. It has been reported that a heterozygous plant with about 50% non-viable pollen may be caused by a chromosomal translocation or inversion in its genome [30]. Plants with reciprocal translocation was considered to produce unbalanced gametes due to the formation of tetravalents during meiosis and/or the absence of entire chromosome arms [13, 31]. Hu et al. reported that transgenic plants mediated by *Agrobacterium* are heterozygosis for the T-DNA integration [17]. A similar phenomenon was observed in the *yl* line. The wild type loci around the ISs except IS2 could be successfully amplified in the *yl* genome. IS2 and TB was located around the 40-kb homozygous deletion region (from TB to IS2) and the wild type loci around IS2 and TB could not be amplified in the *yl* genome (Additional file 1: Figure S3). This revealed that *yl* line with was heterozygosis for the T-DNA integration and chromosomal translocation. Thus, less viable pollens were observed in *yl* line due to the complex chromosomal translocations (Fig. 1). Since the pollens from the same WT were used for the crosses, the reduced seed vigor of *yl* × WT compared to WT × WT and C11 × WT revealed that the development of gynoecia also be affected by the chromosomal translocations in *yl* (Fig. 1).

### Comprehensive analysis of the genome of T-DNA mutants are required to identify the mutated genes

T-DNA transformants obtained also have been shown to contain chromosomal rearrangements, including translocation, deletion and inversion [14]. Similarly, integration of T-DNA into plant genomes based on *Agrobacterium*-mediated plant transformation may also directly interrupt gene structure. However, transgenic plants with chromosomal structure variations or interrupted gene induced by T-DNA integration displayed an unobvious phenotype, because they were always heterozygous for T-DNA insertion [13]. Thus, many potential mutants are easily ignored when identifying T-DNA lines. Concerning the T-DNA lines with chromosomal translocations, pollen viability analysis could be used for preliminary screening [26]. Parent with reciprocal translocation was considered to produce unbalanced gametes due to the absence of entire chromosome arms and these gametes would be lethal [31]. In this study, the *yl* mutant contained lots of non-viable pollens due to chromosomal translocations (Fig. 1c).

The homozygous mutants could be obtained by selfing and then isolated by the unique phenotype. The homozygous mutants with unique phenotype are valuable genetic materials for exploring the function of the mutated gene in forward and reverse genetics. Researchers always detected the T-DNA ISs and analyzed the sequences flanking the T-DNA ISs in the T-DNA induced mutants. However, according to the previous investigates, complex chromosomal translocations or other chromosomal structural variations are frequent in transgenic lines generated by *Agrobacterium*-mediated T-DNA transformation. Therefore, the genes they identified by using the T-DNA insertions site may be responsible for the mutated phenotype or not. The mutant may also be related to the chromosomal structural variations or loss function of genes induced by the structural variations. Thus, it is essential to research the T-DNA insertion mutants by the whole-genome analysis.

### NGS and PacBio sequencing provide a powerful strategy to analyze T-DNA mutants

Insertional mutagenesis provides an effective way to identify novel genes controlling important phenotypes of plants [6]. However, the identification of mutated genes is still difficult to some extent. Traditionally, TAIL-PCR [32], plasmid rescue [33], inverse PCR [34] and adaptor PCR [35] are used for the identification of T-DNA ISs. However, most of these methods can only identify the flanking sequences of T-DNA boundaries. We first used TAIL-PCR to amplify the flanking sequences of T-DNA ISs. We performed one hundred and twenty rounds of PCR with twelve border primers and ten arbitrary degenerate primers. Only two ISs, IS5 (Chr9, 1,671,992) and IS6 (Chr11, 9,184,715) were obtained by using primer pair RP3, AD5 and 2-RP3, AP1, respectively (Additional file 1: Figure S1, Table S5 and Table S6). The two T-DNA amplified were both cleaved at the right border. However, we identified six T-DNA ISs by genome resequencing. We found that most of the T-DNA sequences were not cleaved at the boundaries in the *yl* genome according to the PacBio data (Fig. 3). In addition, complex tandem T-DNA fragments were detected in the *yl* genome. These tandem T-DNA fragments could be in either direct with intact or part of T-DNA fragments. Studies on model plants such as *Arabidopsis* and rice also indicated that multiple T-DNA copies could integrate into one locus [36, 37]. In this case, it is impossible to isolate all the T-DNA ISs using the traditional methods.

Nowadays, NGS is widely used to identify T-DNA ISs by genome resequencing [17]. NGS is more powerful than the traditional methods benefiting from its high throughput [38]. However, NGS can only identify the connections of T-DNA and host genomic DNA owing to its short read length. Recently, PacBio sequencing is developed to offer much longer reads with less accuracy [39]. Thus, the combination of NGS and PacBio can provide accurate and long reads sequence for whole genome analysis of T-DNA mutants. In this study, we successfully identified T-DNA ISs by NGS data and chromosomal rearrangements involving three chromosomes by PacBio data (Fig. 2). Therefore, the combination of NGS and PacBio sequencing would be benefit for identifying mutated genes and/or chromosomal structure variations in T-DNA mutants.

## Conclusions

In this study, we identified a birch T-DNA mutant *yl* with reduced viable gametes compared to the control plants. The whole genome analysis of T-DNA ISs revealed that complex chromosomal translocations and deletions induced by multiple T-DNA integration into *yl* genome, which may be responsible for the low viable gametes in *yl*. We found that the multiple T-DNA could be consisted of T-DNA fragments in arbitrary length and direction. Then we summarized T-DNA integration model in the *yl* genome. We also provided a method for the analysis of T-DNA mutants using NGS and PacBio sequencing.

## Methods

### Plant materials

Wild type birch (*Betula platyphylla* × *B. pendula*) WT, *BpCCR1* overexpression line 3 (*yl*), *BpCCR1* overexpression line 11 (C11) were grown in a greenhouse. Leaves of WT, C11 and *yl* were collected and immediately frozen using liquid nitrogen, then stored at − 80 °C for DNA extraction.

### Genome resequencing and data analysis

Genomic DNA of WT and *yl* was sequenced using PacBio platform by Novogene (Nanjing, China). To extract the reads both mapped to pGWB2-*BpCCR1* vector and birch genome, clean reads were first mapped to pGWB2-*BpCCR1* vector using minialign [40] with default parameter. The resulting SAM format files were transformed to BAM format files and extracted using SAMtools [41]. Then the extracted reads were mapped to the birch reference genome assembly [42] by BLAST. The sequences of pGWB2-*BpCCR1* were provided in the Additional file 2: Data S1.

To check the deletion of chromosomal fragments, we first mapped the PacBio reads from *yl* and WT to the birch genome using minialign. We then extracted the reads mapped to Chr2 around TB and IS2, and the reads mapped to Chr9 around IS5. Integrative Genomic Viewer (IGV) software were used to display the change of read coverage.

### Pollen viability assay

Pollen viability of WT, C11 and *yl* were determined using Fluorescein diacetate (FDA) staining. FDA solution was prepared according to Heslop-Harrison et al. [43]. Fluorescent images were obtained with a fluorescence microscope (Axioimanger A1, Zeiss, Gottingen, Germany)*.*

### Measurement of seed vigor

Seeds were collected from the hybridization combinations using WT, C11, *yl* as female parent and WT as the pollen donor, respectively. To determine the 1000 seed-weight, 100 seeds were randomly selected and weighed by an electronic balance in three replications. One hundred seeds of WT × WT, C11 × WT and *yl* × WT were germinated on wetted filter paper in petri dishes in three replications. The dishes were plated in a 25 °C greenhouse. The number of seeds germinated were recorded on 7th day.

### DNA extraction, TAIL-PCR and PCR

Total DNA was extracted from leaves of WT, C11 and *yl* lines using a genomic DNA extraction kit (Tiangen, Beijing, China). The nested sequence-specific primers were designed according to the border sequences of T-DNA, including six right border primers (RP1, RP2, RP3, 2-RP1, 2-RP2, 2-RP3) and six left border primers (LP1, LP2, LP3, 2-LP1, 2-LP2, 2-LP3). Ten arbitrary degenerate primers including primers AP1, AP2, AP3, AP4 provided by TaKaRa Genome Walking Kit (Code No.6108) and primers AD1, AD2, AD3, AD4, AD5, AD6 designed based on previous research [44] were used. The primers sequences used for TAIL-PCR were provided in Additional file 1: Table S1. TAIL-PCR was performed using LA Taq DNA polymerase (Takara, Dalian, China) according to the manufacturers’ instructions. Three specific primers were used with each arbitrary degenerate primer in the first, second and third round of TAIL-PCR. Genomic DNA of WT and *yl* were used as template in the first round of TAIL-PCR, respectively. Products from the first round of PCR were used as templates for a second round of PCR, and products from the second round of PCR were used as templates for a third round of PCR. The cycle parameters and thermal condition for TAIL-PCR are listed in Additional file 1: Table S2.

PCR reaction for the verification of genome resequencing results were performed using KOD FX Neo DNA polymerase (Toyobo, Osaka, Japan). The PCR reaction mixture (50 μL) contained 100 ng of genomic DNA (WT, C11 or *yl*), 25 μL of KOD FX Neo 2 × buffer, 1 μL of KOD FX Neo and 0.2 μmol of each primer. Step-down cycle condition was used for the amplification of long fragments and three-step cycle condition was used for the amplification of short fragments. The PCR conditions are listed in Additional file 1: Table S2 and the primers used for PCR were listed in Additional file 1: Table S3.

### DNA sequencing

The three round amplification products of TAIL-PCR and PCR products were detected by electrophoresis on 1% agarose gel. The bright bands were cut from the gel and purified by the EZNA Gel Extraction Kit (OMEGA, Doraville, GA, USA), then cloned into pGEM-T easy vector (Promega, USA) and sequenced or directly sequenced using an ABI 3730XL sequencer by TsingKe Biological Technology (Harbin, China).

## Additional files

Additional file 1: **Figure S1**. Gel electrophoresis of amplification products in TAIL-PCR. **Figure S2**. Gel electrophoresis of PCR amplification products for sequencing. **Figure S3**. PCR amplification products of wild-type sequences around the breakpoints. **Table S1**. Sequences of primers used for TAIL-PCR. **Table S2**. Cycle parameters and thermal condition for TAIL-PCR and PCR amplification. **Table S3**. Sequences of primers used for PCR. **Table S4**. Sequences of PCR product with primers TB-R and IS3-F. The fragment of Chr2 is in bold and the fragment of Chr8 is underlined. Primers IS3-F on Chr8 and TB-R on Chr2 is in italics. Clone was sequenced using primers M13F and M13R. **Table S5**. Flanking sequence of T-DNA right border amplified with primers RP3 and AD5. The fragment of genomic sequence is in bold. The fragment of the pGWB2 vector is underlined. Primer RP3 is in italics. **Table S6**. Flanking sequence of T-DNA right border amplified with primers 2-RP3 and AP1. The fragment of genomic sequence is in bold. The fragment of the pGWB2 vector is underlined. Primer 2-RP3 is in italics. (DOCX 2428 kb)

Additional file 2: **Data S1.** The sequences of pGWB2-*BpCCR1. (TXT 16 kb)*

## Abbreviations

*CCR1*: cinnamoyl-CoA reductase
DSB: double-stranded break
FDA: Fluorescein diacetate
FST: Flanking sequence tag
IGV: Integrative Genomic Viewer
IS: insertion sites
LB: left border
NHEJ: non-homologous end-joining
RB: right border
TAIL-PCR: thermal asymmetric interlaced-polymerase chain reaction
TB: translocation breakpoint
T-DNA: transfer DNA
