# Supplementary material for: Comprehensive characterization of T-DNA integration induced chromosomal rearrangement in a birch T-DNA mutant
Source: BMC Genomics. 2019 Apr 23;20:311. doi: 10.1186/s12864-019-5636-y (PMC6480916; doi:10.1186/s12864-019-5636-y)
Supplement: Supplementary file 1 — Figure S1. Gel electrophoresis of amplification products in TAIL-PCR. Figure S2. Gel electrophoresis of PCR amplification products for sequencing. Figure S3. PCR amplification products of wild-type sequences around the breakpoints. Table S1. Sequences of primers used for TAIL-PCR. Table S2. Cycle parameters and thermal condition for TAIL-PCR and PCR amplification. Table S3. Sequences of primers used for PCR. Table S4. Sequences of PCR product with primers TB-R and IS3-F. The fragment of Chr2 is in bold and the fragment of Chr8 is underlined. Primers IS3-F on Chr8 and TB-R on Chr2 is in italics. Clone was sequenced using primers M13F and M13R. Table S5. Flanking sequence of T-DNA right border amplified with primers RP3 and AD5. The fragment of genomic sequence is in bold. The fragment of the pGWB2 vector is underlined. Primer RP3 is in italics. Table S6. Flanking sequence of T-DNA right border amplified with primers 2-RP3 and AP1. The fragment of genomic sequence is in bold. The fragment of the pGWB2 vector is underlined. Primer 2-RP3 is in italics. (DOCX 2428 kb) [file 12864_2019_5636_MOESM1_ESM.docx]

**
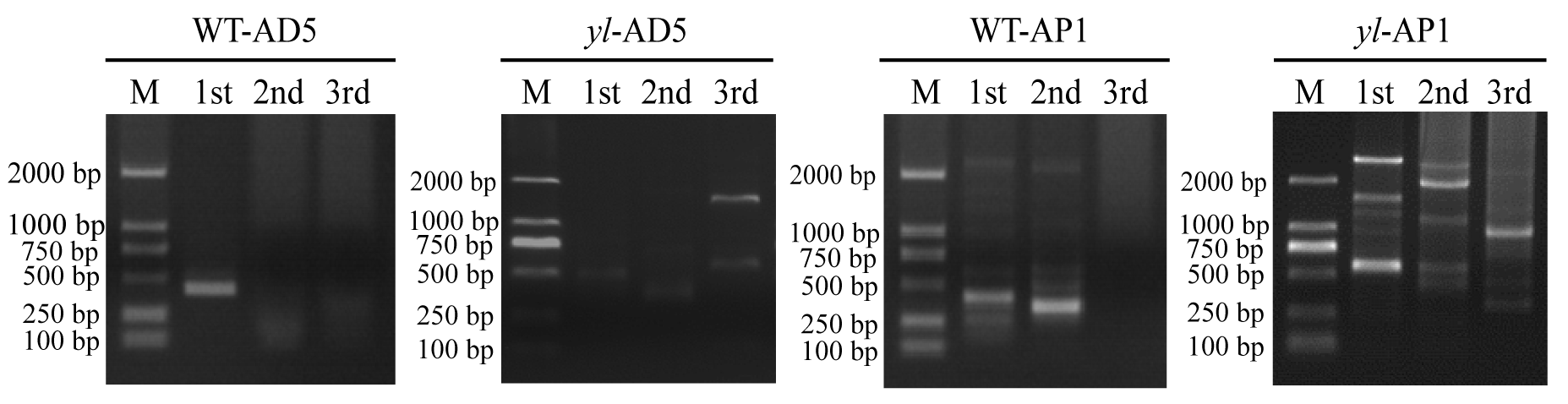
**

**Fig. S1** Gel electrophoresis of amplification products in TAIL-PCR. M. DNA Marker DL2000. 1^st^, the first amplified products. 2^nd^, the second amplified products. 3^rd^, the third amplified product. The nested sequence-specific primers RP1, RP2 and RP3 were used for the amplification of first, second and third round of TAIL-PCR with arbitrary degenerate primer AD5, respectively. The nested sequence-specific primers 2-RP1, 2-RP2 and 2-RP3 were used for the amplification of first, second and third round of TAIL-PCR with arbitrary degenerate primer AP1, respectively.


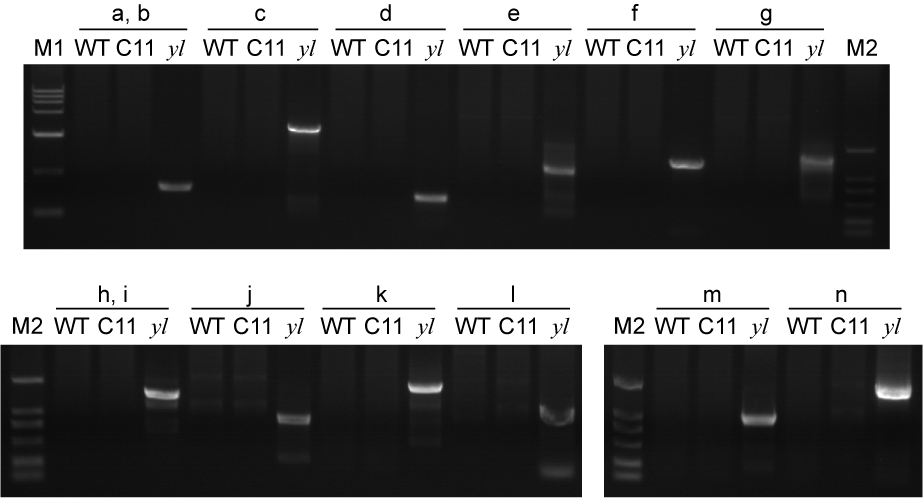


**Fig. S2** Gel electrophoresis of PCR amplification products for sequencing. a, b: primer IS4-R and P5309; c: P10273 and P9182; d: P9925 and P10361; e: P8917 and P4321; f: P6008 and P7464; g: P3054 and IS5-F; h, i: IS2-F and P7033; j: P3328 and P2505; k: P6008 and IS3-R1; l: P7548 and IS3-R1; m: IS6-F and P9925; n: P3328 and IS6-R2.

**
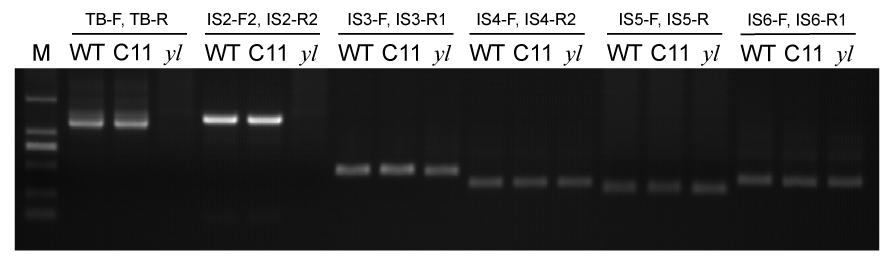
**

**Fig. S3** PCR amplification products of wild-type sequences around the breakpoints. PCR were performed with primer pairs TB-F TB-R, IS2-F2 IS2-R2, IS3-F IS3-R1, IS4-F IS4-R2, IS5-F IS5-R and IS6-F IS6-R1, respectively.

**Table S1** Sequences of primers used for TAIL-PCR

| Name | Primer sequences (5'-3') | Comment |
| --- | --- | --- |
| LP1 | GGGAGATGCAATAGGTCAGGCTCTC | 1348 bp from the left border |
| LP2 | ATATCCACGCCCTCCTACATCGAAG | 1209 bp from the left border |
| LP3 | TCGACAGACGTCGCGGTGAGTTCAG | 1106 bp from the left border |
| 2-LP1 | GGCCGTCGTTTTACAACGTCGTGACT | 604 bp from the left border |
| 2-LP2 | ATAGCGAAGAGGCCCGCACCGATC | 514 bp from the left border |
| 2-LP3 | CCGGCTTTCCCCGTCAAGCTCTAA | 414 bp from the left border |
| RP1 | AAATCACCGGAACCAGAGCCACCA | 1912 bp from the right border |
| RP2 | AGTAACATAGATGACACCGCGCGCG | 1766 bp from the right border |
| RP3 | TCATCGCAAGACCGGCAACAGGA | 1584 bp from the right border |
| 2-RP1 | CCCTTCCCGCTTCAGTGACAACGTC | 602 bp from the right border |
| 2-RP2 | GCACCGGACAGGTCGGTCTTGAC | 503 bp from the right border |
| 2-RP3 | CCGGAGAACCTGCGTGCAATCCA | 373 bp from the right border |
| AD1 | NGACGASWGANAWGAA | - |
| AD2 | NGTCGASWGANAWGAA | - |
| AD3 | AGWGNAGWANCAWAGG | - |
| AD4 | WGTGNAGWANCANAGA | - |
| AD5 | TGWGNAGSANCASAGA | - |
| AD6 | NTCGASTWTSGWGTT | - |

**Table S2** Cycle parameters and thermal conditions for TAIL-PCR and PCR amplification

| PCR  reaction | Cycle number | Thermal condition |
| --- | --- | --- |
| First round of TAIL-PCR | 1  5  1  15  1 | 94 ^o^C 1 min, 98 ^o^C 1 min;  94 ^o^C 30 s, 65 ^o^C 1 min, 72 ^o^C 2min;  94 ^o^C 30 s, 25 ^o^C 3 min, 72 ^o^C 2min;  94 ^o^C 30 s, 65 ^o^C 1 min, 72 ^o^C 2min, 94 ^o^C 30 s, 65 ^o^C 1 min, 72 ^o^C 2min, 94 ^o^C 30 s, 44 ^o^C 1 min, 72 ^o^C 2min;  72 ^o^C 10 min; |
| Second round of TAIL-PCR | 15  1 | 94 ^o^C 30 s, 65 ^o^C 1 min, 72 ^o^C 2min; 94 ^o^C 30 s, 65 ^o^C 1 min, 72 ^o^C 2min; 94 ^o^C 30 s, 44 ^o^C 1 min, 72 ^o^C 2min;  72 ^o^C 10 min; |
| Third round of TAIL-PCR | 15  1 | 94 ^o^C 30 s, 65 ^o^C 1 min, 72 ^o^C 2min; 94 ^o^C 30 s, 65 ^o^C 1 min, 72 ^o^C 2min; 94 ^o^C 30 s, 44 ^o^C 1 min, 72 ^o^C 2min;  72 ^o^C 10 min; |
| PCR (Step-down cycle) | 1  5  5  5  25  1 | 94 ^o^C 2min;  98 ^o^C 10 s, 74 ^o^C 4 min;  98 ^o^C 10 s, 72 ^o^C 4 min;  98 ^o^C 10 s, 70 ^o^C 4 min;  98 ^o^C 10 s, 68 ^o^C 4 min;  68 ^o^C 7 min; |
| PCR (Three-step cycle) | 1  40 | 94 ^o^C 2min;  98 ^o^C 10s, 56 ^o^C 30 s, 68 ^o^C 1 min; |

**Table S3** Sequences of primers used for PCR

| Name | Primer sequences (5'-3') | Comment |
| --- | --- | --- |
| IS2-F | GGGTATGGAGAGATTAGAGAACTAG | Chr2, 26269373, - |
| IS2-R | TGGCGTCCATGTAAATCCCAT | Chr2, 26268938, + |
| IS3-F | GGATCATCTACGCTCCTTGTTTC | Chr8, 5168471, + |
| IS3-R1 | CTATATGTCCTCCTCCCATCCTG | Chr8, 5168906, - |
| IS3-R2 | GCTGATTCTCATCGCACCATAT | Chr8, 5169277, - |
| IS4-F | CAGCAGGGGCATGGAGAGA | Chr8, 17725801, + |
| IS4-R | GGACGGAGGTTCGTGCATC | Chr8, 17726002, - |
| IS4-R2 | GGCAGTCCAAATGTGACCGTTC | Chr8, 17726140, - |
| IS5-F | ACCCACGGGTGGATTTCTTCA | Chr9, 1672356, - |
| IS5-F2 | CCAGTCGCTCCTGGCACATG | Chr9, 1672146, - |
| IS5-R | GACTGGGTTGGGTACCAGTTG | Chr9, 1672141, + |
| IS6-F | GAGAGGAGCGAACCTCGAACTC | Chr11, 9185050, - |
| IS6-R1 | TGGCCCATTGTTCACTCATTTG | Chr11, 9184691, + |
| IS6-R2 | TCTCTCAGTTCGATCTGGCTCTGG | Chr11, 9184372, + |
| TB-F | TCCTTCGAGAAATTACGATGCTAC | Chr2, 26227505, - |
| TB-R | GTTGAAGGGTCAACGGTCAAA | Chr2, 26226093, + |
| P2505 | CTCATGATCAGATTGTCGTTTCC | pGWB2, 2505, - |
| P3054 | TCGTGGCCAGCCACGATAG | pGWB2, 3054, - |
| P3328 | CCCTGATGCTCTTCGTCCAGATC | pGWB2, 3328, - |
| P3895 | CACGATCCTGAGCGACAATATG | pGWB2, 3895, + |
| P4321 | CCACCAGAACCACCACCAGAG | pGWB2, 4321, - |
| P5309 | CAGAACTCGCCGTAAAGACTG | pGWB2, 5309, + |
| P6008 | CCGATGATGCAGAGAAGTTGGTTTGCG | pGWB2, 6008, + |
| P6345 | GGTGTTCTCGCACATGCTGTCATCAC | pGWB2, 6345, - |
| P7033 | TTGAATCCTGTTGCCGGTCTT | pGWB2, 7033, + |
| P7464 | CAAGACAGAGGAACTTGTAAGGAG | pGWB2, 7464, + |
| P7548 | CATGCCGACAGGCATAACTTAG | pGWB2, 7548, - |
| P8917 | GGTGTCGTCCATCACAGTTTG | pGWB2, 8917, + |
| P9028 | CCGCTCGTCTGGCTAAGATC | pGWB2, 9028, + |
| P9182 | TCCGGAAGTGCTTGACATTG | pGWB2, 9182, - |
| P9679 | GTGCCACCTTCCTTTTCTACTGTC | pGWB2, 9679, + |
| P10273 | TGGAACAACACTCAACCCTATC | pGWB2, 10273, + |
| P10361 | TGCTGGGGCAAACCAGCGT | pGWB2, 10361, + |

**Table S4** Sequences of PCR product with primers TB-R and IS3-F. The fragment of Chr2 is in bold and the fragment of Chr8 is underlined. Primers IS3-F on Chr8 and TB-R on Chr2 is in italics. Clone was sequenced using primers M13F and M13R.

| M13F | AGATTGTCTGATGCTTGTTATCGTATTCGCGTGTCGCCCTT*GGATCATCTACGCTCCTTGTTTC*TCGTTACCCTTTTAAAAAATTGGATGAATAAAAAAAATTTGATTAACTATTTTGAATAAGGTATGTGATAGTGTCTATTAACTATTGAATGTGTATTGTCACATTAGTCTAATAAGATAAGAGTGTGAT**TCAATAAAACACGCACAAGACCGTTGAATGGACTTGATTCTCCTTTTTGTTTTGGGCGAGGTATAGGTTTTTGGGTAGGCTTACGTGTTTTTTTCTTACTGGGTCGTGATGTAGGCTATTTAGTATTTTTGGAACGCTCGGGCTGCCGTTGGACTTTTTTTTTTTTTTTTTTTTAACTGAGCCTAACATTTTCATGGGCCAGAATACAAAAACCAAGCAGCGGTCGAATTGACATGGGCTGGGCCGCGAACTTCTGCCTGAAAAATAAGATAATAAAATAAAATAAATTTAGCCAACTAACTATGATCCATATCCAAAGTGAAATAGAAATAATTTATTTTTCGATTAAGATTTTATTTTGTCATATTTAACAATATATATAATGGCACATTATTTAGAAATTTTAAATGACGTGATAATATATACACTATTAAATCTGTAAAATTTAATCTGGAACGTGAAATTATTTCTCATAAACTTCAACCGAAATAATTTTCTTTTTTTCATAAATTCAAACATAACAAATTTTGGAAAATAATGTTTCTCATAATAATGTTAAAGAGAAAATTTCCCATATGAATCTCAAATAATAATAATAATAATAAAAATCAGATCATTAAATTTCTTAACACTCTTTTATTGTAGAGCAATTTACTTTTAAGAATTTAATAAAAAAAACCATATTTATAGGAATGGTTAAAACTCTTATATTCTACCCAAGAGATGCTAATGTATATCACCCATTTCTATATTAGGATTTACTCTACTTAAATTACGGGCTTATGGTATTTGTAACATTCCGATAAAATAATAATAATTTTTTCTTCCCTAGA** |
| --- | --- |
| M13R | TCTCGGCAGACGCGACATCGACTTCGCGTGTCGCCCTT***GTTGAAGGGTCAACGGTCAAA*GAAATCCATTCTACTTTTGACCTGGTTATAGTTTTGACCAAGCTAAATTACTTTTTTGATTATAATTTTCTAAGTTTTGGGTCTCTTTTTTTTTTTTTTTTTTTAAGGTGAACTTCCAATCTTACGTTCAAGAAAATCAAATCATATAAATTTTAAGCGATTACAATATCATGAATTAGTATATCTCTTCCATTCAAATTTTTTTCACAAAAGTCTAAAGCGCCCCCTTTGCCAATAAATGTGCTGCAATCTTTATATCCCTAGTTGTATGCATATGCATCACATACCATGATTGAAGACTATGCAGTATAATTTTTTATTCCTCAATCAAATAATTATTTGGTCTTACTTATAAAATGCTTGTAGTACTATTTTCATAGCATCACCTTCCATAATGACATTGTCACATTGCCTAGGAAACTAGGGAAGAAAAAATTATTATTATTTATCGGAATGTTACAAATACCATAAGCCCCTTATTTAAGTAGAGTAAATCCTAATTATAGAAATGGGTGATATACATTAGCATCTCTTGGGTAGAATATAAGAGTTTTAACCATTCCTATAAATATGGTTTTTTTTATTAAATTCTTAAAAGTAAATTGCTCTACAATAAAAGAGTGTTAAGAAATTTAATGATCTGATTTTTATTATTATTATTATTATTTGAGATTCATATGGGAAATTTTCTCTTTAACATTATTATGAGAAACATTATTTTCCAAAATTTGTTATGTTTGAATTTATGAAAAAAAGAAAATTATTTCGGTTGAAGTTTATGAGAAATAATTTCACGTTCCAGATTAAATTTTACAGATTTAATAGTGTATATATTATCACGTCATTTAAAATTTCTAAATAATGTGCCATTATATATATTGTTAAATATGACAAAATAAAATCTTAATCGAAAAATAAATTATTTCTATTTCAACTTTGGATATGGATCATAGTTAG** |

**Table S5** Flanking sequence of T-DNA right border amplified with primers RP3 and AD5. The fragment of genomic sequence is in bold. The fragment of the pGWB2 vector is underlined. Primer RP3 is in italics.

| **AGATCATGGCAATGGATCCGAGGACCCAAAATTGTTGGACAGTCTAATTCCTTTGCAGCTATGCCTAAGAAATCCAGTCTCGAAAATTCTGAGAATGACTTTGCA**CGCCTAAGGTCACTATCAGCTAGCAAATATTTCTTGTCAAAAATGCTCCACTGACGTTCCATAAATTCCCCTCGGTATCCAATTAGAGTCTCATATTCACTCTCAATCCAAATAATCTGCACCGGATCTGGATCGCTTCGCATGATTGAACAAGATGGATTGCACGCAGGTTCTCCGGCCGCTTGGGTGGAGAGGCTATTCGGCTATGACTGGGCACAACAGACAATCGGCTGCTCTGATGCCGCCGTGTTCCGGCTGTCAGCGCAGGGGCGCCCGGTTCTTTTTGTCAAGACCGACCTGTCCGGTGCCCTGAATGAACTGCAGGACGAGGCAGCGCGGCTATCGTGGCTGGCCACGACGGGCGTTCCTTGCGCAGCTGTGCTCGACGTTGTCACTGAAGCGGGAAGGGACTGGCTGCTATTGGGCGAAGTGCCGGGGCAGGATCTCCTGTCATCTCACCTTGCTCCTGCCGAGAAAGTATCCATCATGGCTGATGCAATGCGGCGGCTGCATACGCTTGATCCgGGCTACCTGCCCATTCGACCACCAAGCGAAACATCGCATCGAGCGAGCACGTACTCGGATGGAAGCCGGTCTTGTCGATCAGGATGATCTGGACGAAGAGCATCAGGGGCTCGCGCCAGCCGAACTGTTCGCCAGGCTCAAGGCGCGCATGCCCGACGGCGATGATCTCGTCGTGACCCATGGCGATGCCTGCTTGCCGAATATCATGGTGGAAAATGGCCGCTTTTCTGGATTCATCGACTGTGGCCGGCTGGGTGTGGCGGACCGCTATCAGGACATAGCGTTGGCTACCCGTGATATTGCTGAAGAGCTTGGCGGCGAATGGGCTGACCGCTTCCTCGTGCTTTACGGTATCGCCGCTCCCGATTCGCAGCGCATCGCCTTCTATCGCCTTCTTGACGAGTTCTTCTGAGCGGGACTCTGGGGTTCGAAATGACCGACCAAGCGACGCCCAACCTGCCATCACGAGATTTCGATTCCACCGCCGCCTTCTATGAAAGGTTGGGCTTCGGAATCGTTTTCCGGGACGCCGGCTGGATGATCCTCCAGCGCGGGGATCTCATGCTGGAGTTCTTCGCCCACGGGATCTCTGCGGAACAGGCGGTCGAAGGTGCCGATATCATTACGACAGCAACGGCCGACAAGCACAACGCCACGATCCTGAGCGACAATATGATCGGGCCCGGCGTCCACATCAACGGCGTCGGCGGCGACTGCCCAGGCAAGACCGAGATGCACCGCGATATCTTGCTGCGTTCGGATATTTTCGTGGAGTTCCCGCCACAGACCCGGATGATCCCCGATCGTTCAAACATTTGGCAATAAAGTTTCTTAAGATTGAA*TCCTGTTGCCGGTCTTGCGATGA* |
| --- |

**Table S6** Flanking sequence of T-DNA right border amplified with primers 2-RP3 and AP1. The fragment of genomic sequence is in bold. The fragment of the pGWB2 vector is underlined. Primer 2-RP3 is in italics.

| **GAGTAAATAGCTCGGACTCGGACAGACCTCGAATTCTAAAACTAAAAGCTCAGTTCCCTTCAGTCTCCCCACATTTTCTTAGCAACCAAACAGAAATCAAACACAGAAAAACAACAAGCAAGGAAAAAGAGGAGACCGGAGAGGAGCGAACCTCGAACTCGGCAGACCTCATCGCGATGGGGATGCAGATGAGGAAGACACCGGCCATGGCAATGGCGGTGTTGAGGCGCCAGTGCTTGGGCCGCCCGTCATCCTCCACACCTTGGCCCGAAAAAATATGATCTGCTCCCCCCCATGGATTGCTATTGCTTTTTCTCTCACTTTCTCTGGTCTTCACCAGCAACCAAAAACCCAATTAGCAGATATGAATCGCTATGCGTATTCGGGTAGGTAGATATGGGATGTGTAAGTTGTGAACCAAGGGTAGGGATAAGGTGGATGTGCTGAATTACAAAAACACCCTCCAAGCTAAGT**AAACACTGATAGTTTAAACTGAAGGCGGGAAACGACAATCTGATCATGAGCGGAGAATTAAGGGAGTCACGTTATGACCCCCGCCGATGACGCGGGACAAGCCGTTTTACGTTTGGAACTGACAGAACCGCAACGTTGAAGGAGCCACTCAGCCGCGGGTTTCTGGAGTTTAATGAGCTAAGCACATACGTCAGAAACCATTATTGCGCGTTCAAAAGTCGCCTAAGGTCACTATCAGCCAGCAAATATTTCTTGTCAAAAATGCTCCACTGACGTTCCATAAATTCCCCTCGGTATCCAATTAGAGTCTCATATTCACTCTCAATCCAAATAATCTGCACCGGATCTGGATCGTTTCGCATGATTGAACAAGA*TGGATTGCACGCAGGTTCTCCGG* |
| --- |
